# A hybrid, iterative approach, to support the development of fit-for-purpose sensor-derived measures

**DOI:** 10.3389/fmedt.2025.1567537

**Published:** 2025-10-30

**Authors:** Belen R. Ballester, Matthew Reaney, Christina Mack, Salma Ajraoui

**Affiliations:** ^1^ Patient Centered Solutions, IQVIA Inc., Barcelona, Spain; ^2^ Patient Centered Solutions, IQVIA Inc., London, United Kingdom; ^3^ IQVIA Inc., Durham, NC, United States

**Keywords:** digital measures, fit-for-purpose DHTs, sensor-derived COAs, DHT, patient-centricity

## Abstract

Digital Health Technologies (DHTs) hold immense potential for transforming drug development. Although innovation in the DHT space has been rapid, the approval process for these technologies remains slow due to fragmented efforts from industry and researchers, as well as regulatory challenges. In this position paper, we propose a hybrid methodology and approach for developing fit-for-purpose DHTs for assessment by integrating both patient-centric and data-centric elements. By emphasizing patient relevance while considering device and data feasibility, we can advance the development of patient-centric digital measures efficiently without compromising measurement precision. Ultimately, this hybrid approach aims to streamline the approval process, foster collaboration among stakeholders, and accelerate the integration of DHTs into clinical practice, thereby enhancing the overall efficiency and effectiveness of drug development.

## 1 Introduction

Digital health technologies (DHTs) offer unprecedented opportunities to transform drug development. Biopharmaceutical companies are leveraging DHTs to enhance existing measures and develop novel digital measures to support drug development and increase our understanding of patient health (1–3). The passive collection of continuous, longitudinal data offers the possibility of a deeper insight into patient experience, extending beyond limited infrequent site visits, informing more nuanced trial endpoints. This approach to clinical trial endpoints also aligns with the growing emphasis on patient-centric care. By focusing on the patient's needs and experiences, healthcare providers can tailor their interventions more effectively. However, the path to approval for digital measures to support clinical trial endpoints has been slow due to siloed and fragmented efforts, limited understanding of the regulatory evidentiary requirements, and a lack of clarity on terminology and definitions (4–6).

**Figure 1:**
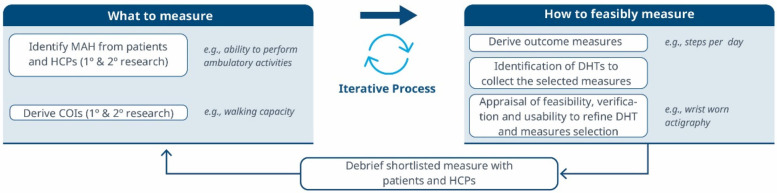
A roadmap for connecting the MAH, the COI and the DHT. An iterative methodology for identifying what to measure and associated DHTs. Debriefing activities evaluate the relevance of the selected measures and technologies, subsequently refining the identification of Concepts of Interest.

**Figure 2:**
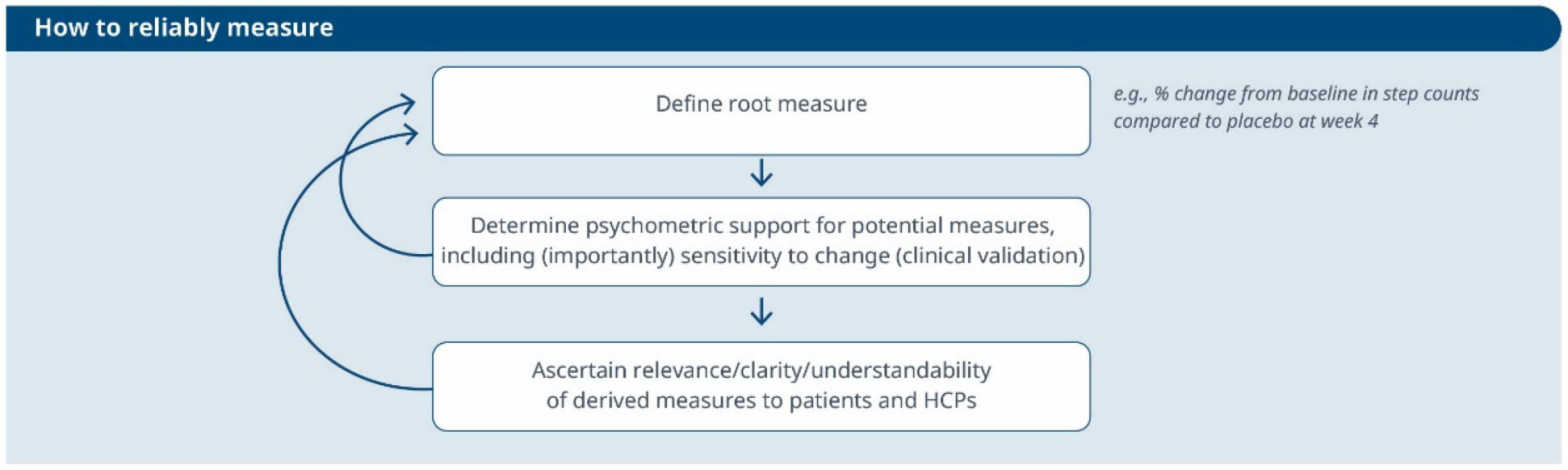
An iterative process to evaluate the reliability of a selected measure and its meaningfulness. This workflow guides the refinement of a root measure intended for use in a clinical trial as an endpoint or biomarker.

**Figure 3:**
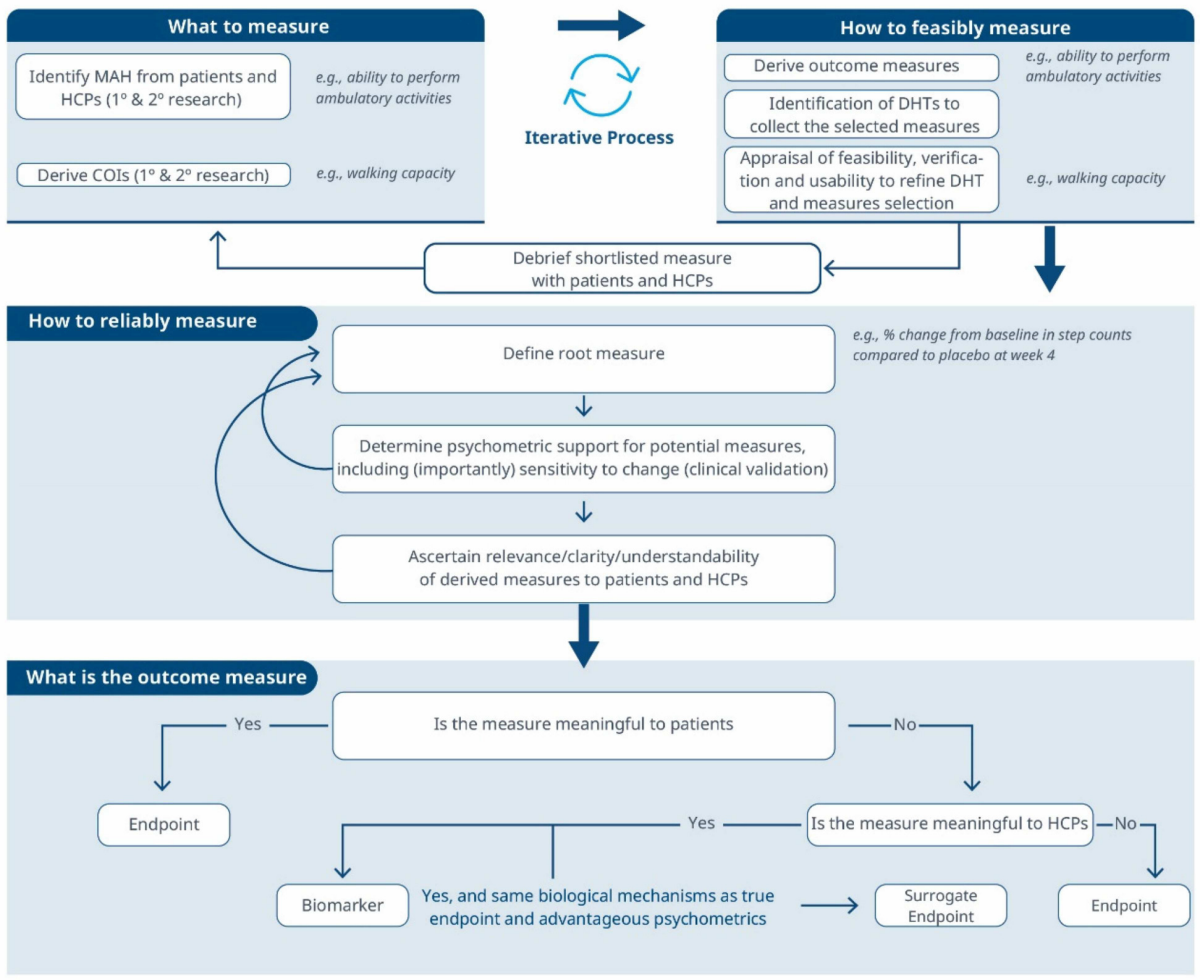
A comprehensive roadmap for selecting and developing novel digital endpoints and biomarkers to be used in drug development. This roadmap includes methodologies for identifying what to measure and establishing how to measure it.

Methods that are used to measure endpoints need to be supported by data to ensure that they are producing reliable information with both internal and external validity. This is essential for approval of the endpoint by regulators and applies to DHTs in the same way as it applies to biomarkers and clinical outcome assessments (COAs). However, while the mechanisms for demonstrating this are well-defined for COA and biomarker endpoints through guidance and reviews and approvals, the newness of current guidance and inconsistent terminology around DHTs have resulted in few reviews/approvals. The era of Patient-Focused Drug Development (PFDD) presents further challenges for DHTs, as it calls for DHT endpoints to be used to support label claims exclusively when the digital measures something relevant and important to patients. This mirrors the environment for COAs, where endpoints gain traction when they align with patient priorities. In contrast, biomarkers often prioritize what can be measured, resonating more with clinicians and scientists than with patients. DHTs, while constrained in their measurement capabilities, operate in real-world environments, and provide additional insights on patients experience which is an advantage over other endpoints. The challenge lies in striking a balance: leveraging DHT data to inform endpoints while maintaining a sound measurement and clinical basis. Some advocate for a patient-led approach, while others favor a data-led approach. We believe that finding a middle ground is crucial to making DHT data appealing to regulators.

To date, the instances of qualification or endorsement of DHTs by the EMA or US FDA are exceedingly rare. The most common cause for rejections of submitted letters of intent and evidence packages to the FDA is the inability to demonstrate the meaningfulness of the digital measure to patients (U.S. Food & Drug Administration, a–c). Therefore, significant gaps exist in the field. The generic methodology recommended by regulators, such as the FDA's PFDD series, for selecting and developing assessment instruments does not adequately address the unique aspects of passive monitoring DHTs. Unlike traditional COAs, which cover a wide range of subjective and objective measures, digital endpoints are constrained by the capabilities of the technology employed to capture them and the feasibility of deploying them within the context of a clinical trial. As a result, researchers must navigate a landscape where technological constraints may impede the comprehensive evaluation of treatment effectiveness and patients' experiences. In this context, following a generic methodology for all COAs may lead to inefficient processes and may not allow to take full advantage of the available technologies.

An alternative sequential process for selecting and developing digital endpoints has been proposed (7). While this approach suggests that technical considerations should follow the identification of the desired measure, it may not be the most efficient in practice. Similarly, the Clinical Trials Transformation Initiative (CTTI) has introduced an “interactive selection tool” to guide the selection of DHTs. This tool emphasizes the importance of identifying relevant measures based on patient input, though it places less emphasis on technical aspects such as feasibility and usability. More recently, and in opposition to the patient-centered perspective, a data-driven approach has been proposed. This perspective is fostered by data scientists who explore the potential of devices and algorithms to capture information, revealing fresh insights and overcoming measurement limitations. In a study by Taylor and colleagues (8), researchers from F. Hoffmann-La Roche AG explored the contrasting patient-centric and data-centric strategies for converting sensor-derived data into digital clinical measures. The article delved into the nuances of these approaches, shedding light on their implications for healthcare. According to the authors, while a patient-centric outcome “uses patient insights to summarize those sensor features that are optimally relevant to patients’ functioning in everyday life”, a data-driven measure refers to “a digital outcome measure that uses a data-driven approach to summarize those sensor features maximally sensitive to the concept of interest”. Both approaches have pros and cons. The patient-centered approach ensures relevance to patients and supports label claims when measures are valid. However, it's inefficient because it neglects technical feasibility until late in the process. On the other hand, the data-driven approach is more efficient but risks creating a digital biomarker unsuitable for endpoints. Moreover, it's methodologically vulnerable due to its departure from the scientific method and susceptibility to biases like overfitting (i.e., poor generalization to new, unseen data) and p-hacking (i.e., manipulating the data analysis to achieve statistically significant results). Recent research analyzed dozens of frameworks and identified three significant gaps in prior frameworks for assessing digital health interventions: they were not sufficiently adapted to address the unique evidence needs of digital health, lacked specific criteria for evaluating evidence quality, and rarely utilized robust methodologies developed for non-digital interventions (9). These shortcomings underscore the necessity for a more tailored and rigorous approach to effectively evaluate digital health solutions.

Drawing from the limitations of these approaches and examining the differences in the development processes for conventional COAs and DHT-passive monitoring COAs, we propose to combine elements of the patient-centric and data-driven approaches to build a holistic and efficient methodology to develop digital measures that are valid, reliable, and meaningful to patients and HCPs. We call this the hybrid approach.

## 2 Hybrid data-and-patient-centric methodology for developing novel digital measures

A hybrid approach integrates elements from both patient-centric and data-centric approaches to develop and validate novel digital measures. While maintaining a patient-centered focus, and starting from what is meaningful to patients, it also acknowledges the importance of considering device and data feasibility early on.

Following the COA approach and V3 framework, we recommend starting the evidence generation journey by identifying Meaningful Aspects of Health (MAHs) and Concepts of Interest (COIs) to patients through research. Afterward, appropriate measures are selected in alignment with COIs, ensuring that the relevant digital assessment instruments measure the COI that matters to patients. It is important to clarify that with the term “digital assessment instrument” (see Table 1) here we refer to the combined DHTs and derived measures that can potentially be used in a clinical trial (e.g., an actigraphy device estimating the time a day that the patient spends in performing moderate to vigorous physical activity). In theory, DHTs and derived measures are independent of each other. However, in practice, often they are closely intertwined. DHT manufacturers not only own the device but also the algorithms that transform the device's raw data into digital measures. For this reason, we define “digital assessment instruments” as the combination of the device and the derived measure. However, in scenarios where the device (i.e., data collector) and the algorithm (i.e., data transformation instrument) come from different DHTs, we can indeed envision a digital assessment instrument as a system composed of multiple instruments working together.

**Table 1:** Terminology and definitions.

Terminology	Definition	Example
Meaningful Aspect of Health (MAH)	Aspect of a disease that a patient (a) does not want to become worse, (b) wants to improve, or (c) wants to prevent (7)	Ability to perform ambulatory activities
Concept of Interest (COI)	A simplified or narrowed element of an MAH that can be practically measured (7)	Lower extremity strength
Outcome Measure	Specific measurable characteristics of the disease that evaluate the MAH as defined by the COI (7)	Steps per day
Endpoint	Precisely defined variable intended to reflect outcomes of interest that are statistically analyzed to address a particular research question (7)	Absolute change in total daily step counts from baseline, compared to placebo, at week 6
Fit-for-purpose DHT	A conclusion that the level of validation associated with a DHT is sufficient to support its context of use. It involves evaluating both its form (i.e., design) and function(s) [i.e., distinct purpose(s) within an investigation] (13)	Use of the ActiMyo Sensor to Assess 95th Centile of Stride in Duchenne Muscular Dystrophy (3)
Clinical Outcome Assessment (COA)	The FDA defines a clinical outcome assessment (COA) as “a measure that describes or reflects how a patient feels, functions, or survives”.	The four types of COAs are: clinician-reported outcome (ClinRO), observer-reported outcome (ObsRO), patient-reported outcome (PRO), and performance outcome (PerfO).
V3 framework	Framework to guide development of digital measures (15)	The V3 framework has three components: • Verification • Analytical Validation • Clinical Validation
Digital Health Technology (DHT)	Technology using computing platforms, connectivity, software, and sensors for health care and related uses, from applications in general wellness to applications as a medical device.	Wearable device such as actigraphy
Digital assessment Instrument	Tool or method used by healthcare professionals, patients, patient's caregivers, or researchers, to collect information and evaluate aspects of a patient's health, functioning, or condition.	Using the Actigraph LEAP to assess physical activity using MVPA data (10)

In this methodology, the connection between MAH, COI and digital assessment instruments will inform the choice of endpoint; whether it is based on a digital biomarker, or a digital endpoint. While digital endpoints capture data related to how a patient feels, functions, or survives, biomarkers are objective and quantifiable characteristics of biological processes and do not necessarily reflect a patient's experience or overall sense of well-being (17). A DHT can serve as both a digital biomarker and a digital endpoint. For example, in a Phase 3 trial conducted by Bellerophon, the FDA endorsed a digital measure of the time spent in Moderate to Vigorous Physical Activity (MVPA) as a primary endpoint for subjects at risk of pulmonary hypertension associated with pulmonary fibrosis (10). This endorsement was based on the measure's clinical meaningfulness. However, if the same MVPA measure were used to predict hospitalization risk in asthmatic patients, it would be considered solely as a predictive biomarker with limited proven relevance to the patients. In the hybrid methodology, various workflows may emerge. For instance, when the association between the MAH, the COI and the digital measure is not firmly established at the early stages, the digital measure can continue its validation process with the goal of developing a biomarker.

We recognize that the order in which evaluation components are considered to develop a novel digital endpoint impacts the outcome. It is crucial to begin with an unmet need rather than an unfounded urge to advance digital health or to use specific digital tools. Initiating the process by actively listening to patients, identifying the most important health-related matters, and evaluating assessment needs within the clinical context is the only way to be aligned with regulators, and the most effective methodology to guarantee patient-centric progress. Delaying assessment of feasibility aspects toward the end of the process can be highly inefficient. In many cases, this approach results in a list of measures that, while proven highly relevant to patients and clinicians, remain unattainable. For instance, results from concept elicitation activities may reveal that sleep quality is what matters most to patients with Chronic Obtrusive Pulmonary Disease, however a proper assessment using DHTs may require the collection of high-resolution EEG recordings, which in most cases would be costly, highly operationally complex, a burden to patients, and generally unfeasible. For this reason, in the hybrid approach, we advocate for a highly iterative Patient-Centric approach, a customized framework that balances patient-centricity and technological feasibility (Table 2). This approach orchestrates research activities towards two connected objectives: identifying what needs to be measured (i.e., the measure) and determining how to measure it (i.e., the digital solution).

**Table 2:** Regulatory guidance in the context of digital measures.

Evidentiary component (extracted from FDA guidelines)	What does it mean in the context of “digital assessment instruments”
1- Concept of Interest (COI) Assessment (Component A): The COA should assess the specific health concept of interest. This ensures that the measure aligns with the intended purpose.	Definition of the unmet need and the rational for using DHTs (e.g., Use of remote monitoring to overcome large severity fluctuations in a specific symptom and improve the precision of the estimates).
2- Comprehensive Coverage (Component B): The selected COA should capture all essential aspects of the COI. It must address the full scope of the concept being measured.	Qualitative evidence from concept elicitation interviews, supported by a conceptual framework consisting of a conceptual model and a measurement model.
3- Understanding Instructions (Component C): Respondents should comprehend the COA's instructions and items as intended by the measure developer. Clear communication is crucial.	Evidence on acceptability aspects (including usability) supporting that the DHT can collect reliable data from the target population.
4- Minimal External Influence (Component D): COA scores should not be significantly affected by factors unrelated to the COI. The measure must focus on the concept of interest.	Feasibility studies (e.g., analysis of patterns in missing data) and complementary psychometrics supporting the construct (e.g., divergent validity).
5- Appropriate Scoring Method (Component E): The method used to score responses in the COA should be suitable for assessing the COI accurately.	Evaluation of the algorithm used to transform data into an endpoint (e.g., accuracy, precision, specificity, AUC). In some cases, it may not apply, such as when no comparator is available.
6- Correspondence to Patient Experience (Component F): COA scores should reflect the specific health experiences related to the COI. This ensures relevance to patients.	Psychometrics supporting the construct (e.g., convergent validity, and known-groups analysis) and qualitative data supporting meaningfulness (e.g., patients’ narratives).
7- Sensitivity to Change (Component G): COA scores should detect clinically meaningful changes within patients over time concerning the COI. Sensitivity is essential.	Psychometrics supporting the ability to detect change. E.g., reliability and responsiveness to change.
8- Interpretability (Component H): Differences in COA scores should be interpretable and clearly communicated in terms of their impact on patient experiences.	Identification of measurable ranges and signal to noise ratios (e.g., MDCs, MCIDs). Qualitative data (e.g., exit interviews) may provide complementary evidence.

## 3 Demonstrating that a digital measure matters to patients and HCPs

As mentioned earlier, regulators are encouraging DHT researchers to look to the guidance on PFDD and COA as a starting point to understand evidentiary expectations for endpoints measured using DHTs. These guidances put a lot of emphasis on showing that COAs are relevant to patients. This has traditionally been done through demonstration of content validity (at least in part). Although this term is commonly used it is also misused, and the new FDA framework has not included this term, instead opting for a framework that says explicitly that the COA should assess a health concept of interest that is important to patients (component A) in a comprehensive manner (component B), and that the score from that COA should reflect the COI well (components E & F) and not be significantly affected by other factors (components C and D) (see Table 2). All of these are also relevant to DHTs and the measures derived from them, but the process may be different and there is therefore some ambiguity and uncertainty. For instance, using a smartwatch to collect total sleep time can support label claims regarding the health concept “sleep quantity”. In contrast, the number of awakenings may not be enough to support claims on “sleep quality”, since other aspects of sleep quality (e.g., feeling rested) would not be captured. These measures may not fully resonate with patients but can significantly aid clinical decision-making. For instance, digital biomarkers fall into this category, and provide valuable information about a patient's health status, disease progression, or response to treatment, even if patients themselves may not fully understand or appreciate their significance.

Considering these limitations, we propose a selection and development methodology to collect the necessary evidence supporting the connection between MAH, COI and digital assessment instruments. This methodology employs a patient-centric iterative approach at two critical stages: first when proving the connection between the MAH, the COI, and the digital measure (stage 1) and when developing the digital measure (stage 2) when clinical validity has been established and the digital measure has become a “root endpoint”. The process involves answering three key questions at each stage: (1) what to measure, (2) feasibility of measurement, (3) reliability of measurement. In detail:

1. **What to measure (see Figure 1)?**
  1. **Identify MAHs and derive COIs:** Understanding the MAHs is a crucial starting point. Patient feedback and narratives provide valuable context and firsthand experiences related to the disease. Once this foundation is established, researchers have two primary avenues for pinpointing relevant COIs. In the first approach, stakeholders are consulted (e.g., using the Delphi Method) to narrow down specific elements of the MAH that can be practically measured. The second method involves delving into existing literature. By conducting a systematic search, researchers uncover published evidence related to patient experiences. In addition, healthcare professionals (HCPs) offer valuable insights that enhance the researchers’ understanding of specific health concepts and related measures. This exploration identifies signs, symptoms, and impacts, supporting the creation of a conceptual model of the disease. The conceptual model of disease has long been a starting point for regulators to ensure that sponsors (drug developers) are measuring what matters to patients and HCPs, rather than just things that they think their product will change.
2. **How to feasibly measure?**
  1. **Derive outcome measures:** Researchers draw insights from patient quotes and HCP feedback and leverage information gleaned from previous clinical trials with the objective of defining methodologies to operationalize the selected COI.
  2. **Identification of DHTs:** This step involves a complete literature review focused on the target population, with the goal of mapping identified COIs to specific digital assessment instruments. By evaluating the historical use of these measures and related instruments in previous clinical trials, researchers gain valuable insights into their appropriate context of use.
  3. **Appraisal of feasibility, verification, and usability to refine DHT and measures selection:** This step entails a detailed analysis for each identified “instrument”. Researchers assess the coverage of symptoms and impact concepts, paying particular attention to any overlapping areas. The evaluation of each instrument is established based on predefined criteria, including COI coverage, verification, usability, and regulatory approval, among other factors. Please notice that with the term “instrument” here we refer to DHTs and derived measures that can potentially be used in a clinical trial (e.g., an actigraphy device estimating the time a day that the patient spends in performing moderate to vigorous physical activity).
  4. **Debrief shortlisted digital measures with patients and HCPs:** At this stage, operationalization involves more than just selecting prioritized digital measures. It also entails bridging the “what” and the “how” by evaluating the connection between the MAH, the COI, and the chosen digital measures. However, the methodology to prove this triad connection remains unclear. While patient interviews and other qualitative approaches are commonly used, they may be insufficient in scenarios where the digital measure is complex and challenging to explain to patients. Researchers in the field are grappling with finding the most suitable approach and will likely need regulatory guidance to establish a method. We propose a patient-centric iterative approach, which involves debriefing the shortlisted digital measures directly with patients. The ultimate goal is to refine measure selection and shortlist digital measures that have a stronger connection with the MAH and the COI. However, it's important to note that certain measures are particularly relevant and applicable to a healthcare professional audience. These measures may focus on aspects that directly impact clinical decision-making (e.g., core temperature as a predictor for developing cytokine release syndrome), even if they don't fully resonate with patients. For instance, biomarkers fall into this category. In such cases, we believe that the connection between the MAs, COIs, and digital measures should be discussed with HCPs.
    This process follows that recommended for regulatory use of COA data (14, 16).
3. **How to reliably measure (see Figure 2)?**
  1. **Define the root endpoint:** Once an instrument (DHT and digital measure) has been identified, the next step is to define an endpoint that efficiently captures change while maintaining good psychometrics. A “root endpoint” is a fundamental endpoint from which various endpoints can be derived. For example, a root endpoint could be defined as the change in a patient's total number of awakenings detected during night hours from baseline on weekday 1 to weekday 15. An endpoint, on the other hand, is a specific metric or result derived from one or more root endpoints. It is used to assess the effectiveness of an intervention or the progression of a condition. For instance, an endpoint could be derived from the root endpoint mentioned above by examining the change in a patient's average number of awakenings during night hours from baseline in week 1 to week 4, excluding weekends. In this derived endpoint, we applied two modifications: (1) the time window was extended to align with the assessment schedules of the trial while ensuring sufficient power to detect change, and (2) the aggregation method was modified to average the number of awakenings per week instead of using a daily timeframe, thereby improving the reliability and accuracy of individual estimates. Several aspects need to be considered at this stage. For example, Demeyer et al. showed that when estimating measures of physical activity, the standardized response means were greater when more days of assessment were included and when weekends were excluded from the analysis (11). Understanding how to standardize the data analysis of a digital measure is crucial for defining a root endpoint and deriving other endpoints that can adapt flexibly to the context of use. There are seven components to consider:
    a. Aggregating Data: How should we aggregate the data? Should we use daily averages, weekly summaries, or some other approach?
    b. Measurement Frequency: How frequently should we measure? Daily, weekly, or at specific intervals?
    c. Timing of Measurements: When should we measure? Is there an optimal time of day or specific points in the intervention period?
    d. Duration of Measurement: How long should we measure? Should we collect data for a fixed duration or until specific criteria are met?
    e. Baseline Definition: How do we define the baseline? Is it a pre-intervention measurement or a reference point within the intervention period?
    f. Effect Calculation: How do we compute the effect? Is it a change from baseline, a relative improvement, or some other metric?
    g. Handling Missing Data: How should we manage missing data? What imputation methods or sensitivity analyses should we employ?
  2. **Determine psychometric support for potential measures**, including analytical and clinical validation (sensitivity to change, as well as reliability, convergent and divergent validity; see Components F and G in Table 2). For instance, when identifying an endpoint for measuring improvements in step counts using a wrist-based actigraphy device in subacute stroke patients, several considerations come into play. After reviewing relevant literature and conducting a meta-analysis of the instrument's psychometrics across different contexts, we may find that improvements become visible only after 6 weeks post-intervention. To enhance precision, continuous data collection during waking hours, weekly aggregation (excluding weekends), and calculating change ratios within each subject may be recommended. Grounding on these results, the “root endpoint” could be defined as the ratio of weekly (weekdays only) averages in step counts over an individual's baseline at 6 weeks post-treatment.
  3. **Ascertain relevance/clarity/understandability of derived measures to patients and HCPs:** As part of the final step, it is essential to discuss the “root endpoints” with patients or HCPs in connection to the seven components outlined earlier. This involves gathering qualitative data to demonstrate that measuring an effect in these specific endpoints is relevant to patients or HCPs (see component H in Table 2). For example, we can explore whether improvements in ambulatory activities (as indicated by step counts) on weekdays matter to patients. Endpoints that are adapted to the operational limitations and characteristics of the study can be derived later from the “root endpoint”, taking into account any constraints, such as the need for data aggregation.

The process can yield different outcomes (see Figure 3). If a measure proves meaningful to patients, it may lead to creating a digital endpoint for label claims. Alternatively, if meaningfulness isn't established but the measure is valuable to HCPs, it becomes a digital biomarker. In rare cases, this biomarker may align with the same biological mechanisms as the true endpoint and have favorable psychometrics, potentially supporting label claims as a surrogate endpoint.

## 4 Discussion

**Figure 4:**
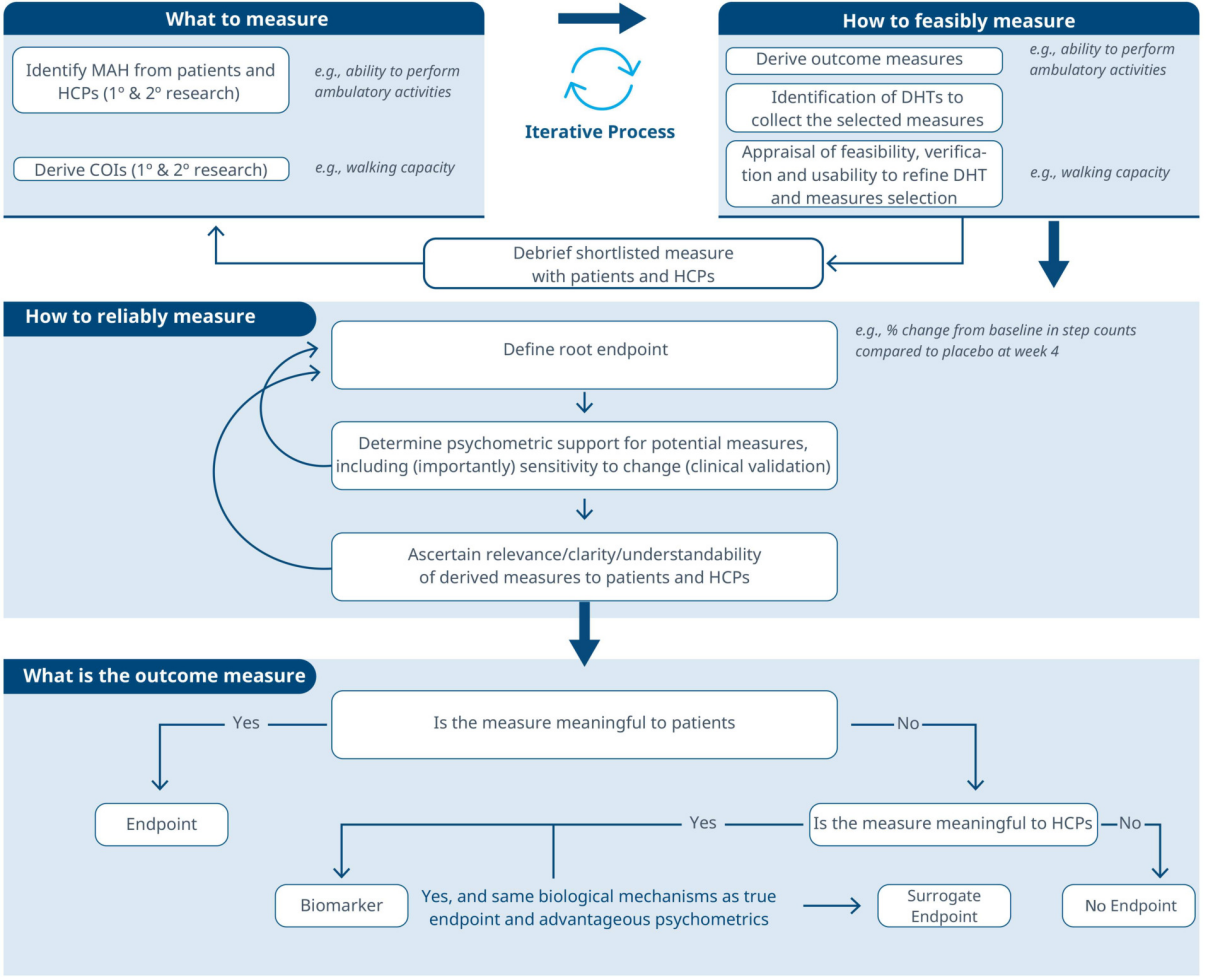
Required evidentiary thresholds depending on the novelty of the digital collection (DHT) or measure.

The hybrid approach offers promise in digital health assessment, by combining patient relevance, feasibility assessment, and psychometrics analyses, researchers can advance patient-centric progress while maintaining measurement precision and robustness. The process begins with identifying what matters to patients, to guide the selection of a digital solution with robust psychometrics and subsequently assessing the meaningfulness of derived endpoints. To establish a robust connection between the three layers of description of the clinical assessment (the MAH, the COI, and the digital instrument), an iterative exploration process is employed. This involves investigating “what” (the MAH and the COI) and “how to measure” (the digital instrument) to create a cohesive link. Evidentiary thresholds will be higher if focusing on novel digital collection (DHT) and/or measures; see Figure 4.

An alternative strategy, which we term the “opportunistic hybrid”, reverses this order. This modification is not done intentionally at the very beginning of the process, but due to convenience. In the opportunistic hybrid, researchers identify a digital measure with excellent psychometric properties and then evaluate its meaningfulness to patients. This approach was successfully employed by the ActiMyo team to gain acceptance from the European Medicines Agency (EMA) for deploying the stride velocity 95th centile (SV95C) endpoint in Duchenne Muscular Dystrophy (12). The data-driven approach is central to this methodology; therefore, it can lead to heterogeneous methods and critical methodological flaws if not carefully considered. For example, the issue of circular analysis (also known as “double-dipping”) arises from reusing data or analyzing it multiple times in the same study. This practice may artificially inflate the significance of findings in *post-hoc* known groups analyses and overestimate the strength of relationships between clinical measures. To address this, researchers are encouraged to use transparent methods and different datasets (such as splitting training and testing datasets) to ensure independent validation of their findings. Another significant methodological flaw in the “opportunistic hybrid” approach is potential experimenter bias. Researchers' prior knowledge of results (e.g., digital biomarkers with excellent clinical performance) and assumptions about measured constructs can influence the identification of health-related aspects and concepts reported by patients and other stakeholders. To mitigate this bias, the team conducting the content validity study should remain blind to digital biomarker validation outcomes. This approach, while convenient, is neither as scientifically robust nor as comprehensive or tied to patient experience or relevance as the proposed hybrid approach.

In the context of the hybrid approach, iterative engagement with regulators throughout the development process is crucial. A well-defined process with planned interactions and decision points will allow researchers to maintain flexibility and optimize efficiency. This approach supports the rapid adaptation of our research roadmap to achieve varying levels of validity and produce digital endpoints, digital biomarkers, or surrogate endpoints as needed. It's therefore essential to set realistic expectations for different scenarios, considering technological and operational limitations. For instance, in some cases, establishing a connection between the MAH, COI and the digital measure may be only partially achievable. This limitation arises when the target digital measure does not fully encompass the entire health concept but only addresses a specific aspect. Consequently, we should plan evidentiary requirements by hand with regulators and in alignment with these limitations.

## 5 Conclusions

This article underscores the potential of a hybrid approach for developing digital measures by integrating patient-centric perspectives with data-driven considerations. This allows researchers to advance patient-centric progress in equal part to focusing on measurement precision and robustness through iterative engagement with regulators. Additionally, the article emphasizes methods for setting realistic expectations, addressing technological limitations, and describes a framework to support the connection between MAH, COI, and digital measure triads.

## Data Availability

The original contributions presented in the study are included in the article/Supplementary Material, further inquiries can be directed to the corresponding author.

## References

[1] Izmailova ES Maguire RP McCarthy TJ Müller MLTM Murphy P Stephenson D. Empowering drug development: leveraging insights from imaging technologies to enable the advancement of digital health technologies. Clin Transl Sci. (2023) 16(3):383–97. 10.1111/cts.13461 36382716 PMC10014695

[2] Sarraju A Seninger C Parameswaran V Petlura C Bazouzi T Josan K Pandemic-proof recruitment and engagement in a fully decentralized trial in atrial fibrillation patients (DeTAP). NPJ Digit Med. (2022) 5(1):80. 10.1038/s41746-022-00622-9 35764796 PMC9240050

[3] Servais L Camino E Clement A McDonald CM Lukawy J Lowes LP First regulatory qualification of a novel digital endpoint in duchenne muscular dystrophy: a multi-stakeholder perspective on the impact for patients and for drug development in neuromuscular diseases. Digit Biomark. (2021) 5(2):183–90. 10.1159/000517411 34723071 PMC8460979

[4] Boehme P Hansen A Roubenoff R Scheeren J Herrmann M Mondritzki T How soon will digital endpoints become a cornerstone for future drug development? Drug Discov Today. (2019) 24(1):16–9. 10.1016/j.drudis.2018.07.001 30009955

[5] Landers M Dorsey R Saria S. Digital endpoints: definition, benefits, and current barriers in accelerating development and adoption. Digit Biomark. (2021) 5(3):216–23. 10.1159/000517885 34703976 PMC8490914

[6] Leyens L Northcott CA Maloney L McCarthy M Dokuzova N Pfister T Why language matters in digital endpoint development: harmonized terminology as a key prerequisite for evidence generation. Digit Biomark. (2024) 8(1):1–12. 10.1159/000534954 38222479 PMC10783888

[7] Manta C Patrick-Lake B Goldsack JC. Digital measures that matter to patients: a framework to guide the selection and development of digital measures of health. Digit Biomark. (2020) 4(3):69–77. 10.1159/000509725 33083687 PMC7548919

[8] Taylor KI Staunton H Lipsmeier F Nobbs D Lindemann M. Outcome measures based on digital health technology sensor data: data- and patient-centric approaches. NPJ Digit Med. (2020) 3(1):97. 10.1038/s41746-020-0305-8 32715091 PMC7378210

[9] Silberman J Wicks P Patel S Sarlati S Park S Korolev IO Rigorous and rapid evidence assessment in digital health with the evidence DEFINED framework. NPJ Digit Med. (2023) 6(1):101. 10.1038/s41746-023-00836-5 37258851 PMC10232404

[10] Nathan SD Flaherty KR Glassberg MK Raghu G Swigris J Alvarez R A randomized, double-blind, placebo-controlled study of pulsed, inhaled nitric oxide in subjects at risk of pulmonary hypertension associated with pulmonary fibrosis. Chest. (2020) 158(2):637–45. 10.1016/j.chest.2020.02.016 32092321

[11] Demeyer H Mohan D Burtin C Vaes A Heasley M Bowler RP Objectively measured physical activity in patients with COPD: recommendations from an international task force on physical activity. Chronic Obstr Pulm Dis. (2021) 8(4):528–50. 10.15326/jcopdf.2021.0213 34433239 PMC8686852

[12] Servais L Yen K Guridi M Lukawy J Vissière D Strijbos P. Stride velocity 95th centile: insights into gaining regulatory qualification of the first wearable-derived digital endpoint for use in duchenne muscular dystrophy trials. J Neuromuscul Dis. (2022) 9(2):335–46. 10.3233/JND-210743 34958044 PMC9028650

[13] FDA-2021-D-1128. (2023). *Digital Health Technologies for Remote Data Acquisition in Clinical Investigations*. Available online at: https://www.fda.gov/media/155022/download (Accessed August 15, 2025).

[14] FDA. (2023). *PFDD3 Guidance Document*. Available online at: https://www.fda.gov/media/173587/download (Accessed August 15, 2025).

[15] Goldsack JC Coravos A Bakker JP Bent B Dowling AV Fitzer-Attas C Verification, analytical validation, and clinical validation (V3): the founation of dertermining fit-for-purpose for biometric monitoring technologies (BioMeTs). NPJ Digit Med. (2020) 3:55. 10.1038/s41746-020-0260-4 32337371 PMC7156507

[16] Reaney M. Chapter 4 & 6. In: *Using Patient Experience Data to Evaluate Medical Interventions: Generating, understanding and using patient experience data within and alongside clinical trials.* s.l.:s.n., pp. 43–56 & 75–88 (2023).

[17] Strimbu K Tavel JA. What are biomarkers? Curr Opin HIV AIDS. (2010) 5(6):463–6. 10.1097/COH.0b013e32833ed177 20978388 PMC3078627

