# Correction: A hybrid, iterative approach, to support the development of fit-for-purpose sensor-derived measures

**DOI:** 10.3389/fmedt.2025.1756558

**Published:** 2026-01-16

**Authors:** Belen R. Ballester, Matthew Reaney, Christina Mack, Salma Ajraoui

**Affiliations:** 1 Patient Centered Solutions, IQVIA Inc., Barcelona, Spain; 2 Patient Centered Solutions, IQVIA Inc., London, United Kingdom; 3 IQVIA Inc., Durham, NC, United States

**Keywords:** DHT, digital measures, fit-for-purpose DHTs, patient-centricity, sensor-derived COAs

A Correction on A hybrid, iterative approach, to support the development of fit-for-purpose sensor-derived measures By Ballester BR, Reaney M, Mack C and Ajraoui S (2025). Front. Med. Technol. 7:1567537. doi: 10.3389/fmedt.2025.1567537

**Error in figure/table**

Wrong content

There was a mistake in Figures 3, 4 as published. During publication the same figure was added twice instead of the right figure. The corrected Figures 3, 4 appears below.

**Figure 3:**
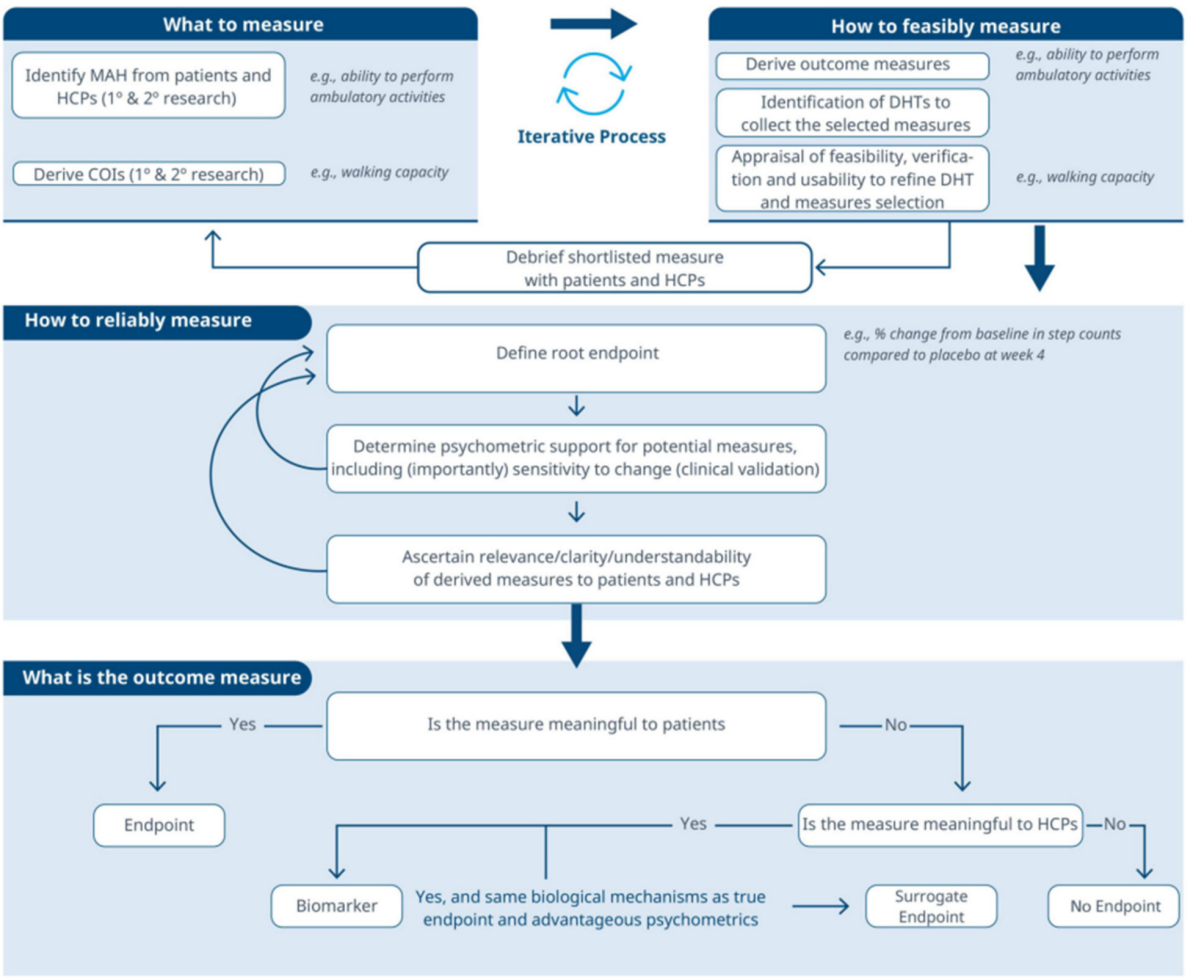


**Figure 4:**
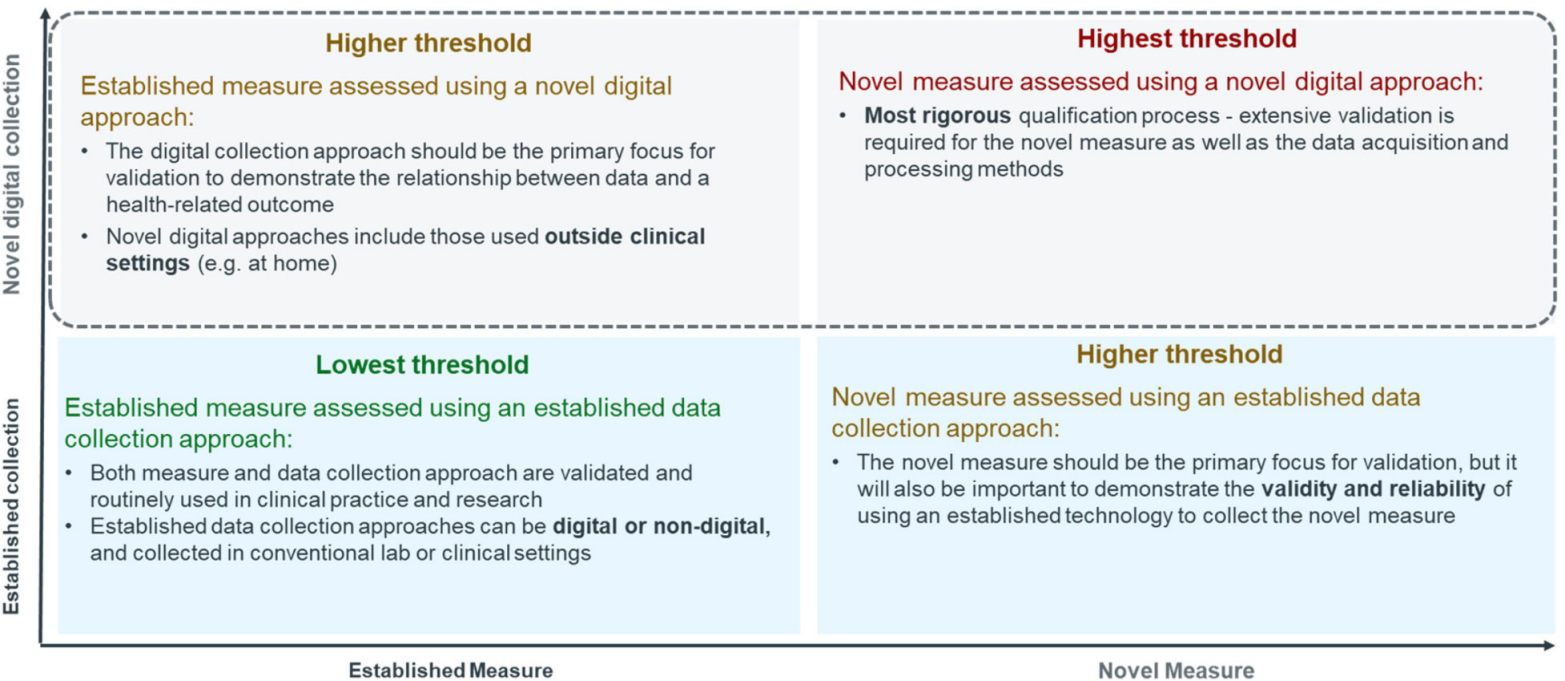


Figure 3 should be as per below (which is current Figure 4 in the published article):

Figure 4 should be as per below:

The original version of this article has been updated.

